# Supplementary figures and images for: An amino-terminal fragment of apolipoprotein E4 leads to behavioral deficits, increased PHF-1 immunoreactivity, and mortality in zebrafish
Source: PLoS One. 2022 Dec 15;17(12):e0271707. doi: 10.1371/journal.pone.0271707 (PMC9754248; doi:10.1371/journal.pone.0271707)

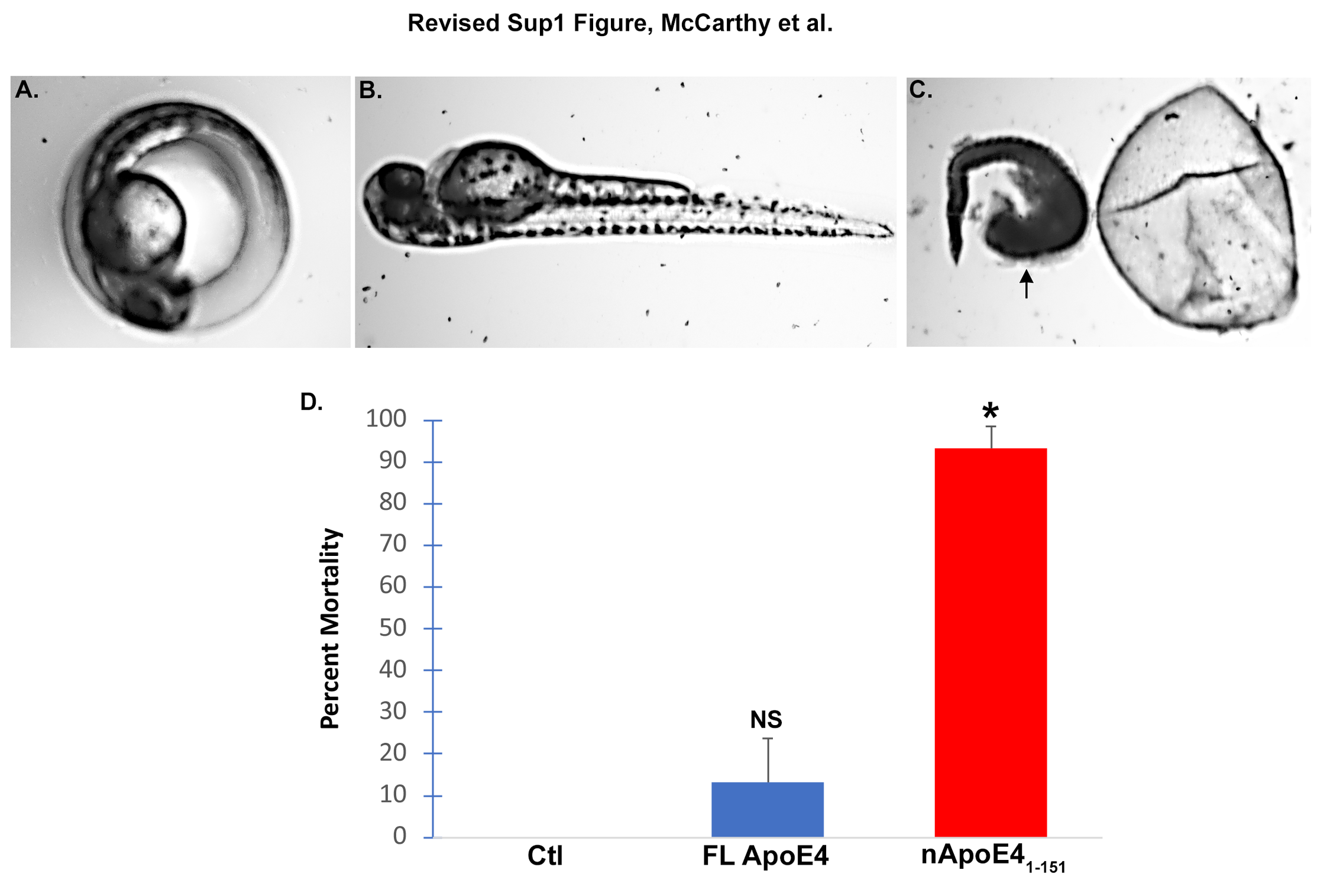

Supplement: S1 Fig — Representative images displaying morphological effects following treatment with either 50 μg/ml full-length ApoE4 (B) or nApoE41-151 (C) indicated that full-length ApoE4 showed little effects on morphology as compared to non-treated controls (A). Embryos treated with full-length ApoE4 resulted in 13/15 fish had exiting their chorion. In contrast, significant morphological effects (arrow, C) and mortality were observed in the nApoE41-151-treatment group (D). Data are representative of three independent experiments (N = 5 per group). No significant difference was observed in mortality between non-treated controls and full-length ApoE4 (p = 0.19). Significant mortality (93%) was observed in the nApoE41-151-treatment group (p = .000076). (TIF) [file pone.0271707.s001.tif]
