# Supplementary material for: An amino-terminal fragment of apolipoprotein E4 leads to behavioral deficits, increased PHF-1 immunoreactivity, and mortality in zebrafish
Source: PLoS One. 2022 Dec 15;17(12):e0271707. doi: 10.1371/journal.pone.0271707 (PMC9754248; doi:10.1371/journal.pone.0271707)
Supplement: Attachment — Submitted filename: Response to Reviewers.doc [file pone.0271707.s006.doc]

**Response to the Reviewers and Academic Editor: We would like to thank the Reviewers for taking the time to evaluate our manuscript and provide helpful suggestions and comments in improving the manuscript. All changes to the revised document are shown in the red, marked up version. We believe the suggestions and comments raised by the Reviewers have significantly improved the quality of the manuscript for which we are grateful.**

Article title: *An amino-terminal fragment of apolipoprotein E4 leads to behavioral deficits, increased PHF-1 immunoreactivity, and mortality in zebrafish*

**Comments from the Editor:**

1. Please ensure that your manuscript meets PLOS ONE's style requirements, including those for file naming. Response: Using the formatting guidelines provided by PLOS ONE, we have made sure to use the proper style requirements to the best of our knowledge.
2. We note that the grant information you provided in the ‘Funding Information’ and ‘Financial Disclosure’ sections do not match. When you resubmit, please ensure that you provide the correct grant numbers for the awards you received for your study in the ‘Funding Information’ section. Response: We have ensured that the grant information now matches.
3. Please remove any funding-related text from the manuscript and let us know how you would like to update your Funding Statement. Response: We have deleted funding-related text from the manuscript and have updated our Funding Statement.
4. PLOS ONE now requires that authors provide the original uncropped and unadjusted images underlying all blot or gel results reported in a submission’s figures or Supporting Information files. Response: We have no submitted gels or blots as figures in this manuscript, so no raw images are available to submit to the journal.
5. Please include captions for your Supporting Information video files at the end of your manuscript, and update any in-text citations to match accordingly. Response: We have now included captions for our supporting information videos in the revised manuscript and have updated any in-text citations to match accordingly.

**Review 1 points of concern: We appreciate the thoroughness by which the Reviewer examined our manuscript and believe the suggestions given have significantly improved the manuscript. In this manner, we attempted to answer every concern of this Reviewer and the additional experimentation recommended by this Reviewer has in our minds strengthened the manuscript.**

1. The developmental phenotypes seem to reflect a general toxicity rather than organ specific targeting, with edemas, smaller ear, loss of pigmentation. Could the authors check for ApoE4 fragment localization in different organ or cell types like heart or muscle cells? It will help to understand the toxicity development, even more since the embryos did not recover after treatment removal.

Response: We believe this is an excellent suggestion by the Reviewer and have now incorporated new experimental data showing staining within muscle tissue in the tail region of zebrafish embryos (new Fig 7). In this manner we now show a disruption of muscle cell organization following treatment with nApoE4_1-151_ (herein designated the E4 fragment) that is similar to what has previously been reported in the literature for the overexpression of a mutant AChR in zebrafish (Ref 55 of revised manuscript). As the Reviewer suggests, these findings strengthen our previous results and provide a mechanism as to the motor behavior deficits even after removal of the fragment from the media. These new results are described in the result section (lines 393-401) and in the discussion (lines 499-505).

2. At the cellular level, the immunolabeling is not homogeneous regarding the quality of staining. Could the author provide lower magnification of the section in addition to the high magnification? It will help to apprehend the results. In addition, the anti-His- labeling is quite different between the SupFig1 and figure 4 for the same 48 hpf stage, could the authors explain this difference? Response: In response to this concern regarding the quality of staining in our original Fig. 4B, we now present new experimental data showing high magnification of the nuclear labeling of nApoE41-151 following treatment. We believe this data strongly support the nuclear localization of the fragment. Moreover, we now also include new data showing the co-localization of the E4 fragment with a specific nuclear, neuronal marker, NeuN (Fig. 4D). In addition, as suggested by the Reviewer, we now include new experimental data in whole embryo mounted sections at low magnification in order to display overall staining in the entire embryo. These results are presented on lines 313-325 of the revised manuscript. We have also removed the original SupFig1 from the revised manuscript. In general, we consistently observed a more or less punctate pattern of nApoE4_1-151_ labeling that was perfectly consistent to our previous results in BV2 microglial cells (ref 18). The nuclear localization is rather clear, but it would add to have a 3D reconstruction of the optical plans to fully convince of the subcellular location. Response: to answer the Reviewer directly, we did not perform 3D reconstruction of the optical planes to fully show the subcellular location. However, we do have multiple z-stack image analyses that indicate nuclear labeling (data not shown), and we believe our new data shown in Fig 4 showing strong co-localization of the E4 fragment with NeuN, support nuclear localization. Another question to strengthen the important result of co-localization of ApoE4 fragment and Tau PHF1: could the authors provide a quantification of this neuronal co-localization? How many cells could be observed for each embryo? Response: Unfortunately, we did not quantify this potential relationship, but we plan on exploring the phf-1 findings in more detail in a future study.
3. The previous data of the authors have been obtained on microglial cells. What about zebrafish microglial / macrophages presence during the treatment? It can be discussed if no data are available. Response: This is an important point raised by the Reviewer. In fact, during the time-frame of development that we employed zebrafish embryos, it is known that they do not as of yet express fully functional glial cells (Ref 29). This is one reason we focused on neuronal populations in the current study. Additionally, we have previously identified the E4 fragment within neurons of human postmortem AD brain sections. Therefore, we were also interested in exploring whether we could identify nuclear localization in neurons in this study. We have added language addressing this on lines 232-236 of the revised manuscript.

**Minor points**

1-Figure 1 Moderate score should be considered as severe score. Response: We agree with the Reviewer on this point and appreciate this observation. We have now amended Fig 1 to reflect this change.

2- How many sections per embryos have been observed? Response: A minimum of 3 sections per embryo were analyzed. This language was added to the revised manuscript on lines 222-223.

3- Table 1: could the authors provide the reference # of the NeuN antibody, since no mouse polyclonal is found on the catalog. Response: We have now added the exact clone (1B87) that was used in our studies in the revised Table 1.

4- For Touch Escape Response locomotor test, usually the trajectory length and total time of swimming are plotted (Campanari et al 2021 as an example), could the authors provide these parameters? Response: We appreciate this excellent recommendation by the Reviewer. We have included new experimental data whereby the total distance traveled, and total time of swimming are reported (Revised Fig. 6). We also included heat map data from a control and a low-performing nApoE4_1-151_-treated larvae (Fig 6C, bottom panels). Interesting, results for these new data, total swimming distance (p=.08) and swimming time (p=.06) just missed significance as compared to untreated controls. From the supplementary movie, it looks like the embryo is on its back which means it has a vestibular/ear problem. Could the authors have another example of embryo with no abnormality? Response: Our anecdotal observations were that many of the E4-treated fish we tested for TEMR exhibited this behavior of being on their back or side. Because we did not validate this finding statistically in the current manuscript, we are not commenting on this finding in the revised manuscript. However, we are providing an additional raw video showing this behavior following treatment with the E4 fragment.

**Review 2 points of concern: We appreciate the insightful comments that this Reviewer provided and believe those suggestions have improved the manuscript.**

1. My major concern is that the authors have conducted the experiments on 24 hpf, 48hpf and 72hpf zebrafish embryos for modelling a disease like AD. In the introduction section it is mentioned that the majority of AD cases are characterized as late onset AD, only 5% cases comprise of early onset of AD. How can it be concluded if it is AD or any other neurological outcome such as poor outcome of brain injury after treatment of exogenous ApoE fragments or any tauopathy other than AD? Justify. Response: We believe this is an excellent point raised by the Reviewer. The goal of the present study was not to employ zebrafish as a model of AD *per se*, but to test whether a risk factor associated with AD could lead to toxicity or other potential negative consequences in an *in vivo* model. In this manner, the use of wild-type zebrafish embryos was used to extend our previous *in vitro* findings in transformed cells [Ref 16-18]. The primary weakness in our previous published work was the use of transformed, BV2 microglia cells in an entirely *in vitro* model system. These weaknesses can be summarized as relying on data from a single, murine, immortal microglia cells that may not be representative of normal, non-transformed cells. Another potential caveat of our previous studies was the reliant upon *in vitro* model systems to investigate the pathophysiological actions of nApoE4_1-151_. Therefore, the primary goal of the current study was to expand our *in vitro* findings to an intact organism consisting of zebrafish embryos and larvae. Zebrafish have emerged an excellent model organism for studies of vertebrate biology. We have heavily edited the revised manuscript to reflect this idea and to emphasize this was not a model of AD. Our future lab directions are to test the E4 fragment in more appropriate AD models using zebrafish. Changes in the revised manuscript to reflect this new tone given on lines 82-84 of the introduction and lines 430-439 of the discussion.
2. An experiment like this requires very strict adherence to randomization protocols, as the potential for bias is high. I suggest that the authors review the ARRIVE and PREPARE guidelines (PLoS Biol. 2020 <https://doi.org/10.1371/journal.pbio.3000410>; Lab Anim. 2018 Apr;52(2):135-141) to better describe the methodology. Response: We believe this was an excellent suggestion by the Reviewer and have now extensively revised the methods to incorporate the ARRIVE guidelines including how we blinded behavioral studies, average weight, sex etc. as well as providing actual calculations of the numbers of embryos in terms of power of study. We are confident that our methodology was extremely rigorous and our results transparent and reproducible.
3. Line 75-76…References are required to justify the statement. The introduction portion may be improved by including some recently reported research. Some reference the authors should consider are as follows…- Response: We have now provided new references to support the statement in the introduction (Ref 19-21) in the revised manuscript. We have also included the suggested references provided by the Reviewer in the revised manuscript. We thank the Reviewer for providing these important references to us.
4. Line 440…the authors have mentioned that… “our results suggest that they may inhibit BMP signaling”… only pigmentation change was reported here. Please explain. Molecular marker-based studies are required to justify the statement. Response: We agree with the Reviewer on this point and because we have no data to support this statement, all statements regarding BMP signaling have been removed from the revised manuscript.
5. Figure 4 and supplementary figure 1 qualities are not up to mark. Cell boundaries are not distinguishable in some cells. Response: Because the Reviewer had issues with these two figures in terms of quality, we have removed the supplementary Figure from the revised manuscript. In addition, we have completely revised Fig 4 and replaced that data with new data showing crisp, high-magnification images that clearly show nuclear localization of the E4 fragment (revised Fig 4A-C).
6. The manuscript should be read by a native English speaker. Response: All authors including the corresponding author are native English speakers. We have carefully reviewed the manuscript for any typographical and/or grammatical errors. If the Reviewer could be more specific as to specific areas where our English is subpar, we would be happy to revise those sections.
